# Artificially designed synthetic promoter for a high level of salt induction using a *cis*-engineering approach

**DOI:** 10.1038/s41598-024-64537-z

**Published:** 2024-06-13

**Authors:** Sneha Lata Bhadouriya, Arti Narendra Karamchandani, Namitha Nayak, Sandhya Mehrotra, Rajesh Mehrotra

**Affiliations:** Department of Biological Sciences, Birla Institute of Technology and Sciences Pilani, Goa campus, Goa, India

**Keywords:** Abiotic stress, CaMV35S, *Cis*-regulatory elements (CREs), GUS activity, Motif, Salinity stress, Synthetic promoter, Plant biotechnology, Plant stress responses

## Abstract

This work aimed to design a synthetic salt-inducible promoter using a cis-engineering approach. The designed promoter (PS) comprises a minimal promoter sequence for basal-level expression and upstream *cis*-regulatory elements (CREs) from promoters of salinity-stress-induced genes. The copy number, spacer lengths, and locations of CREs were manually determined based on their occurrence within native promoters. The initial activity profile of the synthesized PS promoter in transiently transformed *N. tabacum* leaves shows a seven-fold, five-fold, and four-fold increase in reporter GUS activity under salt, drought, and abscisic acid stress, respectively, at the 24-h interval, compared to the constitutive CaMV35S promoter. Analysis of *gus* expression in stable *Arabidopsis* transformants showed that the PS promoter induces over a two-fold increase in expression under drought or abscisic acid stress and a five-fold increase under salt stress at 24- and 48-h intervals, compared to the CaMV35S promoter. The promoter PS exhibits higher and more sustained activity under salt, drought, and abscisic acid stress compared to the constitutive CaMV35S.

## Introduction

Plant stress is an external (biotic or abiotic) constraint that limits primary production by reducing the photosynthetic rate. Soil salinity is a detrimental stress that decreases plant yield and survival rate^1–3^. Salt stress reduces the average yield by 20–50% in major crop type^4,5^. Accumulation of ions such as Na^+^, Cl^−^, Mg^2+^, SO_4_^2−^, Ca^2+^, CO_3_^2−^, HCO_3_^−^, and K^+^ primarily manifests as an osmotic and ionic shock, which leads to the generation of reactive oxygen species (ROS), resulting in oxidative stress^6–10^.

Plants overcome these conditions through osmolyte production, epigenetic modifications, or changes at transcriptional levels, thereby maintaining the cell’s ultrastructure^11,12^. Stress-responsive mechanisms involve altering gene expression rates of transporters and enzymes. Genes involved in stress response are induced at high levels by transcription factors (TFs) binding to specific *cis-*regulatory elements (CREs) in their promoters. CREs are short sequences (3–25 nucleotides) arranged uniquely in the promoter’s proximal and distal regions or downstream of the transcription start site (TSS). CREs determine TF binding and regulate gene expression^13,14^. The TF binding specificity of CREs depends on their distance from TSS, spacer sequence, copy number, flanking sequences, inter-motif distance, and orientation^15^.

Promoters can be constitutive, tissue-specific, or inducible^16^. A constitutive promoter directs gene expression in all plant tissues at all stages. However, driving transgene expression through a constitutive promoter may lead to epigenetic gene silencing, suboptimal growth, and increased metabolic burden in plants^17^. A tissue-specific promoter specifically expresses the gene in a particular organ or tissue but may be undesirable in combating the broad effects of abiotic stresses. An inducible promoter expresses a gene in response to specific physical, chemical, developmental, or environmental cues, making them ideal for driving the expression of a stress-responsive transgene^18–21^.

Utilizing synthetic inducible promoters for transgene expression has several advantages: (1) the stockpile of natural stress-responsive plant promoters is limited. (2) Synthetic promoters can be designed to reduce the chances of homology-dependent gene silencing (HDGS). (3) Synthetic promoters can be designed to be small in size and, therefore, easily incorporated into DNA constructs. (4) Several studies have indicated that altering aspects like spacing and copy number of CREs can increase the strength and specificity of synthetic promoters compared to native promoters^22–26^.

In this study, we aimed to design a robust salt-inducible synthetic promoter. To this end, the promoters of genes upregulated under salinity stress in different plant species were screened for CREs. The synthetic promoter was designed using high-copy number CREs arranged by their preference in order and inter-motif distances observed in native promoters^27^. Conserved TATA and CAAT boxes were placed upstream of the core promoter element. A synthetic promoter (PS) sized 454 bp was synthesized. Transient and transgenic studies were conducted in *Nicotiana tabacum* and *Arabidopsis thaliana*. The GUS expression levels of the synthetic promoter (PS) and the control CaMV35S promoter were measured under different stress/hormonal conditions.

## Materials and methods

### Identification of salt-responsive genes

The list of genes upregulated under salt stress from different plant species was obtained from the Microarray Expression Atlas (https://www.ebi.ac.uk/gxa/home). Genes having 40% or higher expression in salinity were selected. These genes were confirmed using the Next-Generation sequencing database (https://mpss.meyerslab.org/). A total of two thousand five hundred genes were short-listed for the analysis.

### Screening native promoters for CREs

The sequence 1 kilobase (Kb) upstream from the start site was obtained from the NCBI database (https://www.ncbi.nlm.nih.gov) and delineated as the promoter region. Two thousand three hundred sixty promoters, excluding redundant sequences, were short-listed. CREs in the short-listed promoter sequences were identified through PlantCARE (http://bioinformatics.psb.ugent.be/webtools/plantcare/html/)^28^ and PLACE (http://www.dna.affrc.go.jp/htdocs/PLACE/) databases. The location, inter-motif distance, and copy number of CREs were listed.

### Promoter designing

CREs from genes showing 4- to 25-fold higher expression levels under salinity stress were considered for the study. The location, copy number, and spacer distance of the CREs were decided based on their preferences observed in the selected native promoters. Core promoter sequences (TATA and CAAT boxes) essential for transcription were inserted downstream of the CREs. Specific restriction sites (5′*Hind*III and 3′*Bam*HI) were included for cloning purposes.

### Transient expression analysis

#### Cloning

The designed synthetic promoter (PS) construct was ordered from Bioresources Biotech Ltd., Bengaluru, India and amplified with the primer set:

*PS Forward Primer; 5′-TATGCGCCAAGCTTACGT-3′* and

*PS Reverse primer; 5′-GGCGGATCCGGAGGAAGCC 3′*.

The amplicon was inserted in pBI121 vector upstream of the reporter *gus* gene. pBI121 with CaMV 35S was used as control. PS: *gus* construct and CaMV 35S: *gus* were amplified using primer sets PSFP-GURP and CVFP-GURP, respectively:

PSFP (forward primer): 5*′*-TATGCGCCAAGCTTACGT- 3*′* and

GURP(Reverse primer) 5*′*-GACGTCGACTCTAGTAAC-3*′*

CVFP (Forward primer) 5*′*-CCAAGCTTGCATGCATG-3*′*, and

GURP (Reverse primer) 5*′*-GACGTCGACTCTAGTAAC-3*′*

The amplicons were sub-cloned into pBS SK(+) vector for particle bombardment. The small size of the pBS SK(+) vector (2.96 kb) makes it a better alternative for transient expression.

#### Preparation of plant material for particle bombardment

*Nicotiana tabacum* (cv. Xanthi) was used for transient gene expression studies. The plants were grown in a 1:0.5:3 mixture of vermiculite, clay soil, and sand in the greenhouse. Greenhouse conditions were adjusted to 24 °C temperature, 60% humidity, and 16 h day/8 h night cycle. Leaves were harvested from 9-10-week-old plants for transient expression studies. The leaves were sterilised using 70% (v/v) ethanol (1 min) and 0.1% HgCl_2_ (v/v) (30 s), followed by (5x) washing with autoclaved distilled water. After sterilisation, the midrib was removed and small sections of the leaves were cultured on Murashige and Skoog (MS) agar media and maintained at 24 °C with 16/8 h light/dark cycle until bombardment. The bombardment was carried out at 1100 lbs/cm^3^ pressure (Bio-Rad). Post bombardment, the explants were cultured for 48 h on MS plates in a plant growth chamber maintained at 24 °C with a 16-h light/8-h dark cycle.

#### Treatment with Sodium chloride (NaCl), Abscisic acid (ABA), Polyethylene glycol (PEG), Salicylic acid (SA) and Jasmonic acid (JA) stressors for GUS assay

The bombarded leaves were incubated in Hoagland solution medium supplemented with different stressors (100µM SA/50µM JA/100 mM NaCl/50 mM PEG-4000/100µM ABA) and maintained at 24 °C with a 16 h light/8 h dark cycle. Untreated leaves were used as a negative control. The treated and control samples were harvested at different time points (0, 12, 24, and 48 h), snap-frozen in liquid nitrogen and stored at − 80 °C. The *GUS fluorometric assay* was carried out using the bombarded leaf samples^29^. GUS extraction buffer [1 mM EDTA, 50 mM Na_2_HPO_4_ (pH 7.0), 0.1% SLS, 1 mM DTT, and 0.1% v/v Triton X-100] was added to crushed leaves and centrifuged at 4 °C, 13,000 rpm for 20 min. 50 µl of GUS assay buffer [GUS extraction buffer + 2 mM MUG] was mixed with 1 volume of the leaf extract and incubated at 37 °C for 2 h. 900 µl of 0.2 M Na_2_CO_3_ solution was added to stop the reaction. 200 µL of the sample was analysed for relative fluorescence detection of 4-methylumbelliferone (MU) using a spectrofluorometer (365 nm excitation/455 nm emission). MU standard curve was generated and 1 RFU (relative fluorescence unit) value was computed. The concentration of total soluble protein in the leaf sample was tested using the Bradford method^30^ and measured against a Bovine Serum Albumin standard curve. Specific GUS activity was estimated using Bradford assay values for total protein concentration and reported in nmol/min/g protein after being normalised to the protein content of each extract.

### *gus* gene expression analysis in transgenic *Arabidopsis* thaliana

#### Growth conditions

*Arabidopsis thaliana* (ecotype Columbia) (Col-0) seeds were obtained from LEHLE SEEDS Company (Catalog number: WT-02), Texas, USA. For germination on media plates, the seeds were sterilised with 70% (v/v) ethanol, 0.1% mercuric chloride (HgCl_2_) (v/v) for 30 s and rinsed with water. The seeds were placed on 1/2 MS media plates supplemented with 2% sucrose and 0.8% agar and stratified at 4 °C in dark for 3 days. The plates were then shifted to a growth chamber (Daihan Labtech, LGC-5101, India) maintained at 22 °C, 75% relative humidity with 200 µE/m^2^ s light intensity, and 16 h light/8 h dark cycle. After 10–15 days, the plantlets were transferred to pots containing a mixture of perlite, peat moss, and vermiculite in 1:1:1 ratio obtained from Keltech Energies Limited (http://www.keltechenergies.com/horticulture-products.html).

#### Production of stable transgenic plants by floral dip method

*Agrobacterium* tumefaciens (GV3101) were transformed with pBI121: synthetic promoter and pBI121: CaMV35S. A single colony was inoculated in liquid culture with antibiotics (25 μg/ml gentamycin, 50 μg/ml kanamycin, 50 μg/ml rifampicin), and incubated at 28 °C till an OD of 0.8 at 600 nm was reached (log phase). The culture was centrifuged and resuspended in a solution of 5% sucrose and 0.05% Silvett L-77. The floral tissues of *Arabidopsis* were dipped in the solution and then maintained in the dark for 24 h. Seeds collected from mature siliques were sterilized and placed on MS selection plates containing 50 μg/ml kanamycin. The plates were maintained in plant growth chambers at conditions specified previously. Transformants were identified as kanamycin-resistant seedlings with green leaves and well-formed roots. After 15 days, the green and enlarged leaf transformed seedlings were transplanted into pots and cultivated in the growth chamber. The presence of the construct in the extracted genomic DNA was confirmed through PCR amplification using the primers: *PSFP; 5′- TATGCGCCAAGCTTACGT, PSRP; 5'- GGC*GGATC***C****GGAGGAAGCC* (for PS), and *CVFP; 5'CCAAGCTTGCATGCATG, CVRP: 5'CGTGGATCCCTCTCCAAATG)* (for CaMV35S). Positive transgenic lines T_1_ (PS) and T_1_c (CaMV 35S) were self-pollinated to obtain the single insert homozygous T_2_ (PS) and T_2_c (CaMV 35S) lines, respectively.

#### Treatment of transgenic plants with different stress factors

To measure *gus* gene expression levels under different abiotic stress conditions (ABA, salinity, drought), the T_2_ seeds of generated transgenics were germinated and cultured on ½ MS medium for 15 days. The plantlets were transferred to pots containing soilrite and maintained in the plant growth chamber until treatment. Fifteen-day-old and four-week-old transgenics were treated with various abiotic stress agents (ABA, salt and drought). The plant sets were treated with Hoagland’s solution containing 75–300 mM of NaCl/50–200 mM of PEG/100–200 µM of ABA to induce stress. Post-treatment, the plants were harvested at 0, 12, 24, and 48 h. All experimental setups consisted of three biological replicates.

#### GUS Histochemical Staining

Wild-type and transgenic *Arabidopsis* plants treated with NaCl/PEG/ABA were incubated in the GUS reaction mixture [sodium phosphate (Na_2_PO_4_, pH 7.0), potassium ferricyanide (K_3_FeCN_6_), potassium ferrocyanide (K_4_FeCN_6_), Triton-X-100, and 5-Bromo-4-chloro-3-indolyl-d-glucuronide (X-gluc)] for 12 h. The plant tissue was treated with 70% (v/v) ethanol to remove excess pigment. Treated plants were observed for blue colouration under a bright-field microscope (Leica Q500MC, Cambridge, England).

#### Relative *gus* expression analysis by qRT-PCR.

For quantitative real time PCR, total RNA was extracted from treated and untreated transgenic *Arabidopsis* using the Qiagen RNAeasy mini kit (Catalog no. 74104). cDNA was synthesised from the total RNA using Quantitech reverse transcription kit (Qiagen Catalog no. 205311). qRT-PCR reaction mixture contained SYBR Green QPCR Master Mix (Agilent Technologies, Catalog #600882), forward and reverse primers, and 25 ng cDNA. Actin *(ACT2)* was used as the reference housekeeping gene. The primer set used for *ACT2* amplification was: *ACTFP: 5′-AADCACAATCCAAGAGAGGTATTC-3′, ACTRP: 5′-TACATAGCGGGAGAGTTAAAGGTC-3′*. The dissociation curve was analysed for the presence of primer dimers or other nonspecific amplified products. Livak and Schmittgen method was used to check for the relative change in *gus* expression^31^.

## Results

### Screening promoters for CREs involved in salt stress

**Figure 1:**
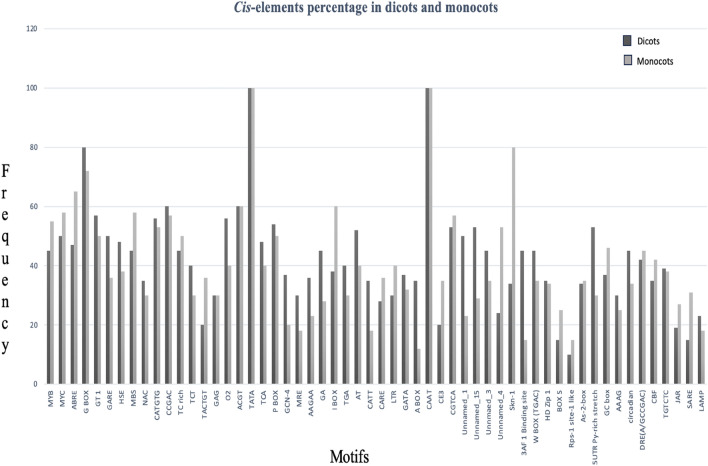
A bar graph representing prominent CREs in the promoters of 2500 salt-induced genes in dicots and monocots. The X-axis represents the CREs and the Y-axis shows the frequency of occurrence in percentage.

2500 genes showing > 40% up-regulation under salt stress were identified from different plant species using the Microarray expression atlas (Supplementary Table 1). The promoter sequences of these 2500 genes were screened to identify high-occurrence CREs using the PLACE and PlantCARE databases. The high-occurrence CREs in dicotyledonous and monocotyledonous plants (listed in Supplementary Table 2) are shown in Fig. 1.

The type, copy number, and spacer distance of the high-occurrence CREs in the different promoters were manually tabulated. Table 1 lists the CREs selected for promoter designing.

**Table 1:** CREs in the designed synthetic salt-inducible promoter PS along with their function and location.

*Cis* element	Sequence	Function	Location
A box	CCGTCC	Abiotic stress responsiveness	-431
ABRE	GCAACGTGTC	Abscisic acid responsiveness	-84, -229, -428
ACGT	ACGT	Abiotic stress responsive element	-80, -110, -149, -241, -389, -441
CCGTCC-box	CCGTCC	Meristem specific activation	-437
CE3	GACGCGTGTC	ABA and VP1 responsiveness	-192
CGTCA-motif	CGTCA	MeJA-responsiveness	-271
MYB	GTGTCG	GA signalling and abiotic stresses (Salt, drought, cold, wounding etc.)	-178, -350
MYC	CGTCACATGCA	ABA stress response	-148
CCGAC	CCGAC	Abiotic stress responsive element	-29, -304
G Box	CACGTA	Light responsiveness	-72, -232
GA motif	AAAGATGA	Light responsiveness	-215
GAG	GGAGATG	Light responsiveness	-263
CATGTG	CATGTG	Abiotic stress responsive element	-230
GT1-motif	ATGGTGGTTGG	Light responsiveness	-273
O2 site	GATGACATGG	Zein metabolism regulation	-96
P Box	CCTTTTG	Gibberellin-responsive element	-290
TCT motif	TCTTAC	Light responsiveness	-64
TGA motif	AACGAC	Stress responsive element	-195, -251
WRKY	TGAC	Heat, drought, salinity and oxidative stress	-322
Unnamed__15	CCTCTCCCGTC	–	-438
Unnamed__3	CGTGG	–	-426, -73

**Figure 2:**
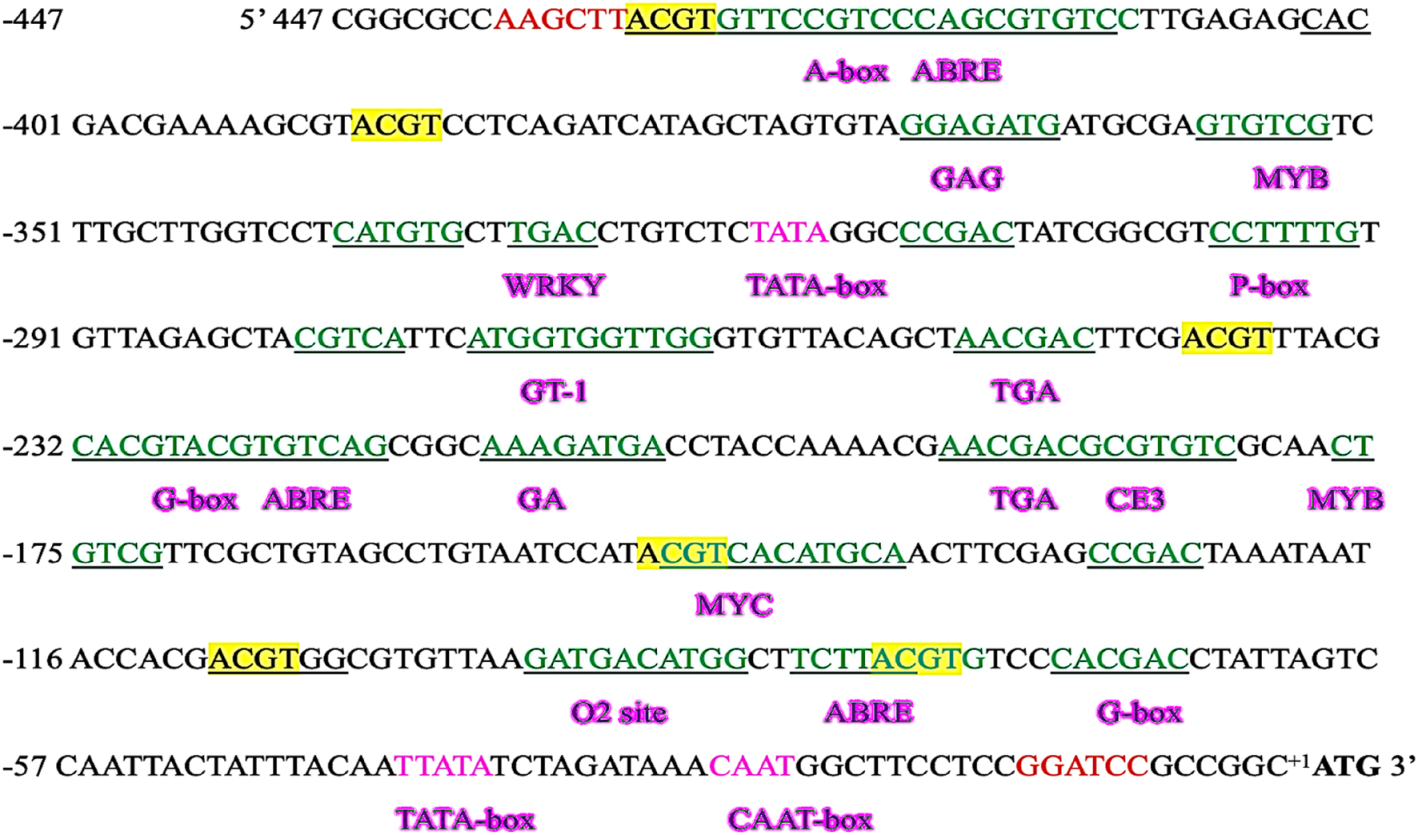
Minimum promoter construct designed for salinity stress induction in plants. The transcription start site (TSS) is indicated in bold letters. The numbers on the left indicate the distance from the TSS. The different stress-responsive CRE sequences are underlined and the ACGT element is highlighted. TATA and CAAT-box sequences are represented in pink. *Hind*III and *Bam*HI restriction enzyme sites are represented in red.

The identified CREs were found to act in Abscisic acid (ABA), Jasmonic acid (JA), and drought stresses. Motifs necessary for transcription initiation like the TATA and CAAT boxes were placed downstream to the proximal promoter region. The synthetic promoter module was flanked with specific restriction sites for cloning purposes. A minimal promoter construct of 454 bps was designed (Fig. 2). The designed promoter was synthesised by Bioresources Biotech Ltd., Bengaluru, India.

### CREs in the synthetic PS and CaMV 35S promoter

CaMV 35S, a constitutive viral promoter which gives a basal level gene expression irrespective of the stress condition, mostly contains single copies of several stress-responsive CREs (Table 2). For high-levels of salt-responsive induction, we incorporated copies of coupling elements like ABRE and ACGT in the PS promoter. Additionally, single copies of several other stress-responsive CREs like A box, CCGAC, GA motif, GAG, P Box, TCT, and CCGAC were introduced (Table 2, Fig. 2). The ACGT element forms the core of Abox to which bZIP protein binds. Many reports have suggested that ACGT core is involved in various biotic and abiotic stress responses. The ABRE is involved in dehydration stress response^32^. Salinity stress ultimately culminates to dehydration stress. ACGT elements form the core of ABRE. MYBs have shown responses in GA signalling and stresses like cold, light, salt, drought, and wounding^33^. The MYC CRE acts in ABA stress-response^34^. ABRE, along with CE3 (coupling element 3), forms an Abscisic acid response complex^35^. WRKY TFs are involved in drought, salt, and heat response^36^. CCGAC and CATGTG functions in drought and salinity stress^37^. GT1 and TGA motifs function in response to salt-induced stress^38,39^.

**Table 2:** List of *cis*-regulatory elements present in the synthetic and CaMV 35S promoter.

Promoter	PS	CaMV 35S
A box	1	–
ABRE	3	1
MYB	2	1
MYC	1	1
CE3	1	–
WRKY	1	–
CCGAC	2	–
G Box	2	1
GA motif	1	–
GAG	1	–
GT1-motif	1	2
O2 site	1	-
P Box	1	–
TCT motif	1	–
TGA motif	2	1
GATA	-	2
ACGT	6	1
CATGTG	1	–

### Transient expression analysis of the designed promoter under different abiotic stress conditions

**Figure 3:**
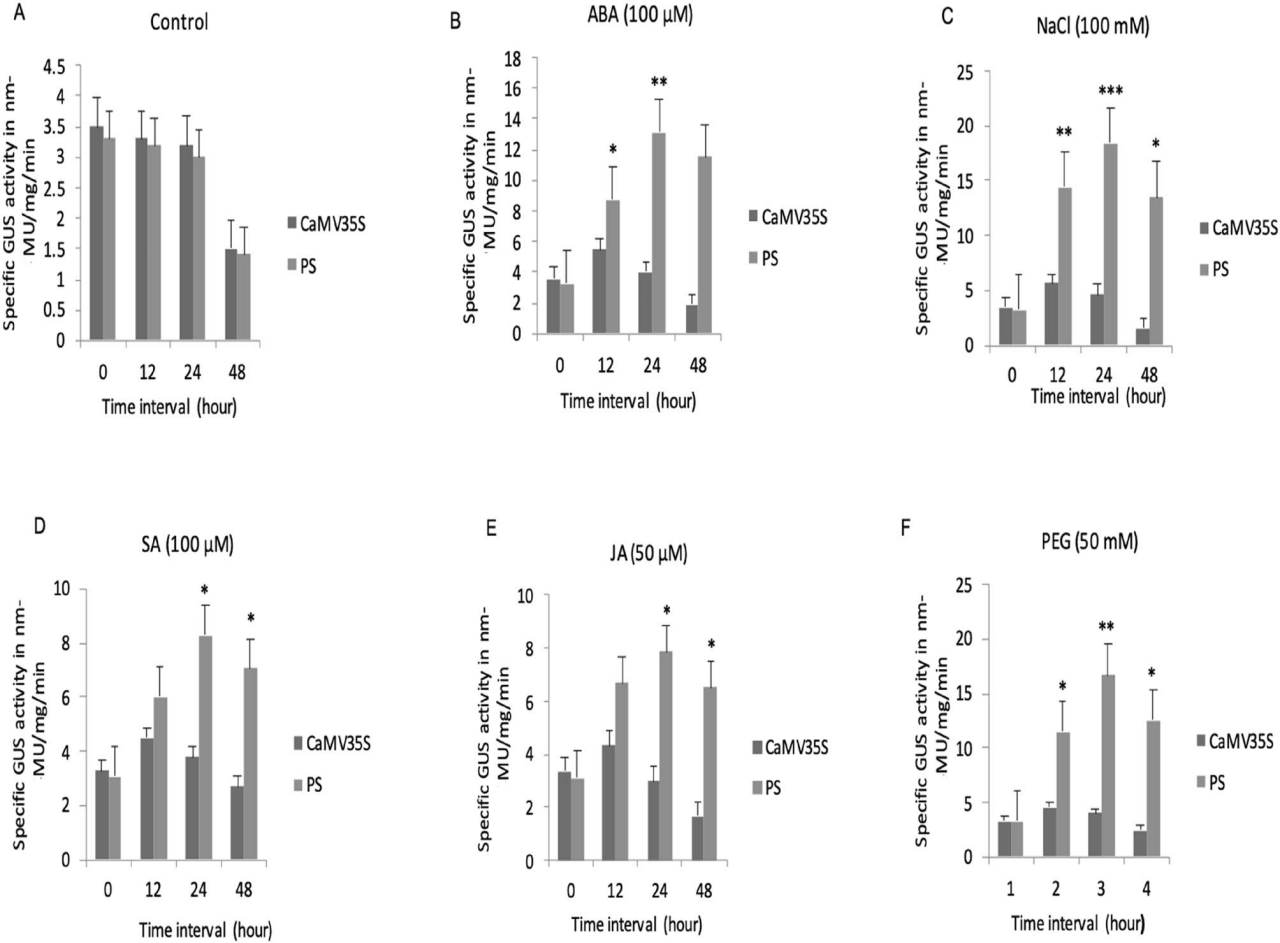
Relative GUS activity of CaMV 35S and PS promoter in transiently transformed *N. tabacum* under different stressors. Representative histogram with standard error from three replicates performed for each time point has been represented. Error bars represent the mean ± standard error in relative abundance of three biological replicates. The statistical significance was determined by student’s t-tests (*P < 0.05, **P < 0.01, ***P < 0.001). The X-axis indicates time interval in hours; Y-axis indicates specific GUS activity. The plots show transiently transformed *N. tabacum* in (**A**) untreated conditions; (**B**) 100 µM abscisic acid (ABA); (**C**) 100 mM NaCl; (**D**) 100 µM salicylic acid (SA); (**E**) 50 µM jasmonic acid (JA); (**F**) 50 mM polyethylene glycol (PEG).

The activity of PS promoter in abiotic stress was measured in comparison to CaMV35S through GUS activity analysis of transiently transfected *Nicotiana tabacum* leaves. The GUS activity was studied under the effect of ABA (100 µM), NaCl (100 mM), SA (100 µM), JA (50 µM), and PEG (50 mM) at 0, 12, 24, and 48 h as shown in Fig. 3. In untreated conditions, the PS and CaMV35S promoters showed similar GUS induction levels (Fig. 3A). However, in all the tested hormonal stress conditions, the PS promoter showed more than two-fold increase in GUS activity, compared to samples containing the CaMV 35S promoter construct (Fig. 3B–F).

High GUS activity was observed at the 24-h time point in PS construct containing samples under all tested stresses (Fig. 3). Compared to CaMV35S, a seven-fold and five-fold increase in GUS activity was observed at the 24-h time point in PS samples under NaCl and PEG treatment (Fig. 3, C, F). A four-fold, one-fold, and 1.5-fold increase in GUS activity was observed in PS promoter containing samples at the 24-h time point when treated with ABA, SA, and JA, respectively, compared to CaMV35S (Fig. 3B, D, E). The induction levels decreased at 48 h under all treatments in CaMV 35S samples but GUS activity in PS samples was still higher compared to the CaMV 35S promoter (Fig. 3). The GUS activity analysis of transiently transformed *Nicotiana tabacum* leaf extracts showed that the synthetic promoter PS induces high levels of gene expression in response to salinity and dehydration stress.

### Expression analysis of gus gene in stable transgenic *Arabidopsis* thaliana plant

#### GUS histochemical analysis of the transgenic T_2_ plants

**Figure 4:**
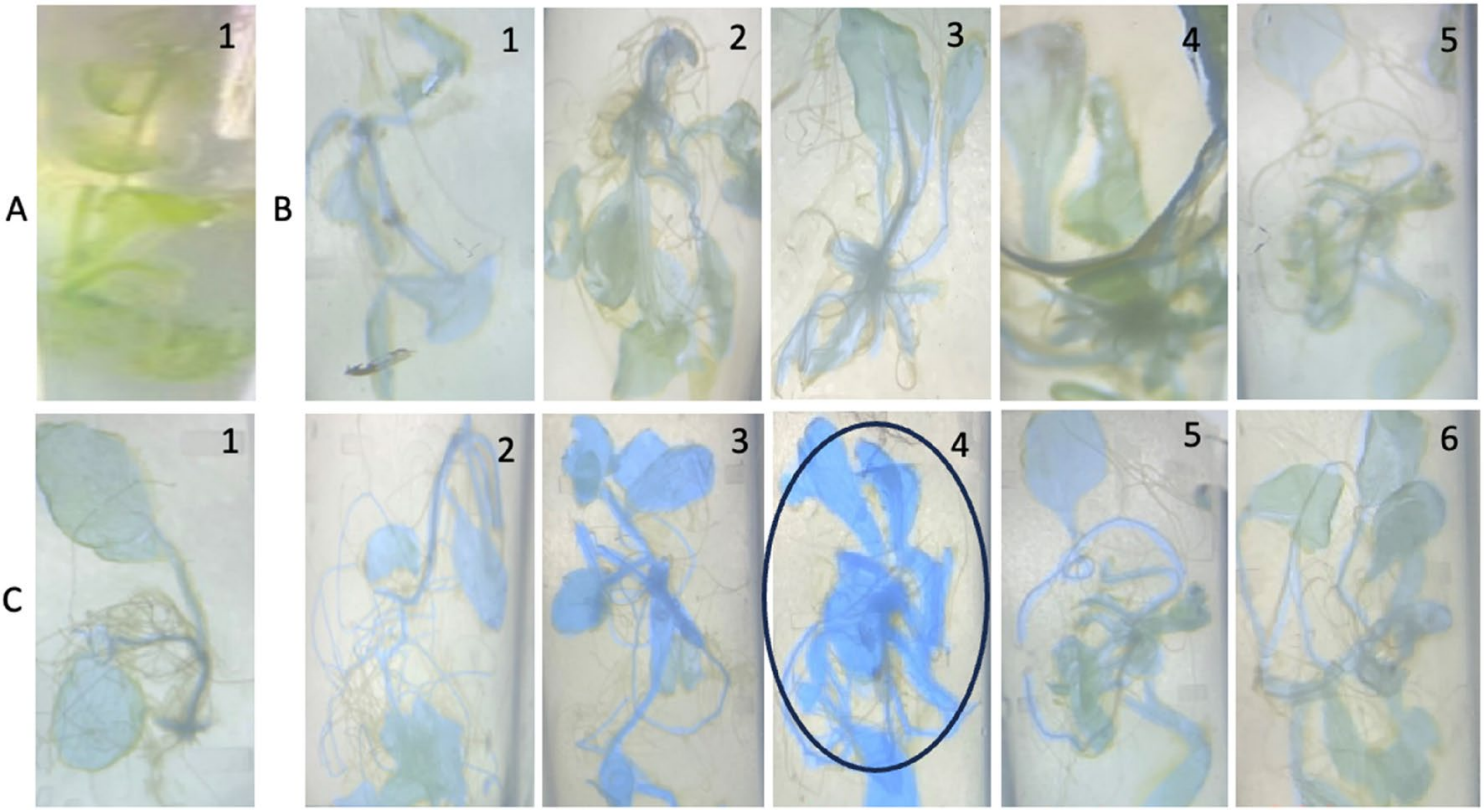
GUS histochemical analysis of 15-day-old transgenic (T_2_) *Arabidopsis thaliana* plants. The plants were observed under a bright-field microscope at 10X magnification. (A) control wild-type plant; (B) transgenic *Arabidopsis* plants with CaMV 35S promoter construct treated with 1, 75 mM NaCl; 2, 100 mM NaCl; 3, 150 mM NaCl; 4, 200 mM NaCl; 5, 250 mM NaCl; (**C**) transgenic *Arabidopsis* plants with PS promoter construct treated with 1, 75 mM NaCl; 2, 100 mM NaCl; 3, 150 mM NaCl; 4, 200 mM NaCl; 5, 250 mM NaCl; 6, 300 mM NaCl.

GUS activity was checked through qualitative histochemical analysis in 15-day-old transgenic plants after treatment of different concentrations of NaCl (Fig. 4). Wild-type plant (negative control) showed no GUS activity (Fig. 4A), T2 plants with CaMV 35S promoter construct showed relatively weak GUS activity (Fig. 4B), in comparison to plants with PS promoter construct (Fig. 4C).

**Figure 5:**
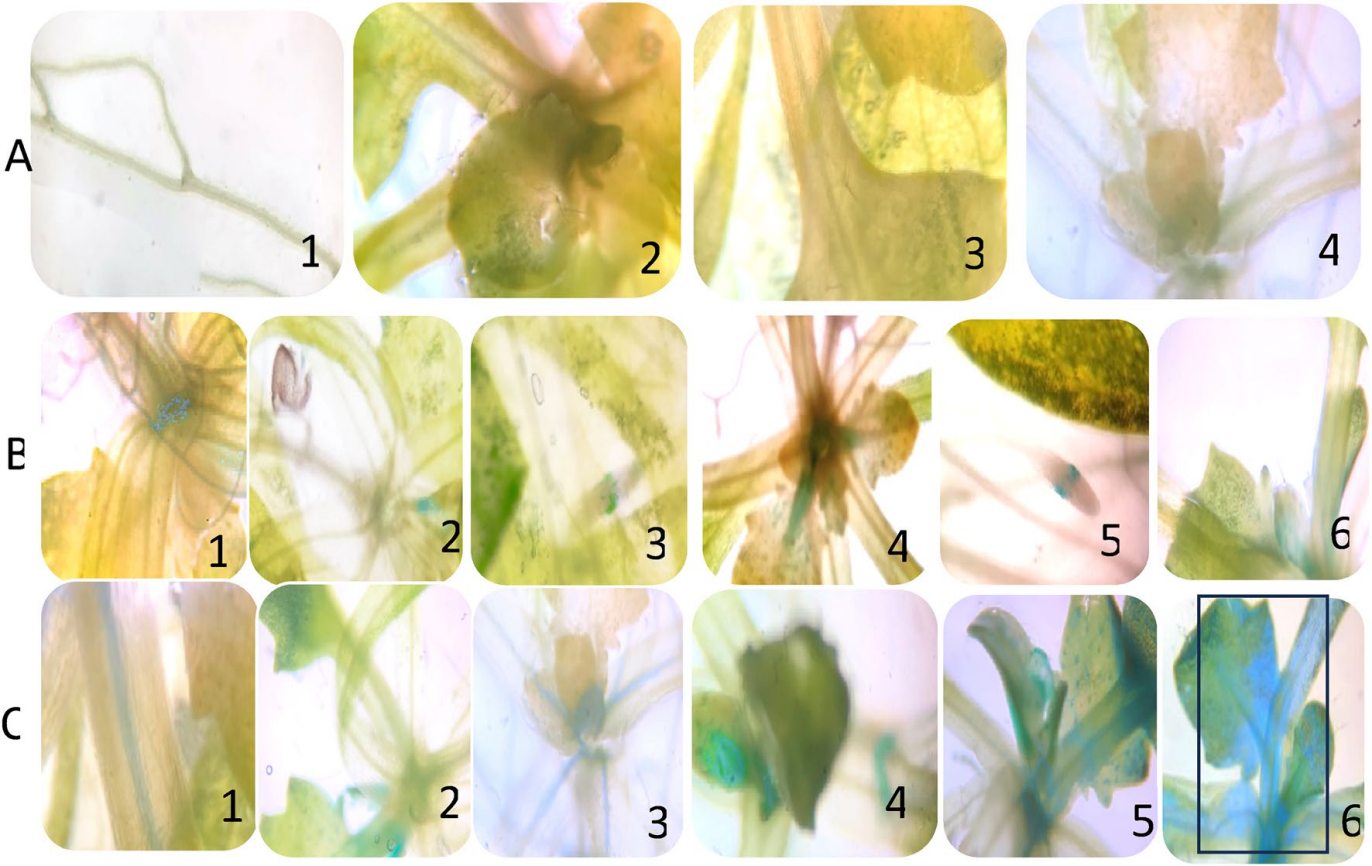
GUS histochemical analysis of 4-weeks-old wild-type and transgenic *Arabidopsis thaliana* plants. The plants were observed under a bright-field microscope at 10× magnification. (A) Wild-type *Arabidopsis thaliana*; (B) T2 transgenic *Arabidopsis thaliana* harboring CaMV35S:*gus* construct treated with 1, 100 µM abscisic acid (ABA); 2, 150 µM abscisic acid (ABA); 3, 50 mM polyethylene glycol (PEG); 4, 100 mM polyethylene glycol (PEG); 5, 100 mM NaCl; 6, 150 mM NaCl; (**C**) T2 transgenic *Arabidopsis thaliana* harboring PS:*gus* construct treated with 1, 100 µM abscisic acid (ABA); 2, 150 µM abscisic acid (ABA); 3, 50 mM polyethylene glycol (PEG); 4, 100 mM polyethylene glycol (PEG); 5, 150 mM NaCl; 6, 200 mM NaCl.

The GUS histochemical assay was also performed in 4-week-old transgenic plants treated with different concentrations of ABA and PEG (Fig. 5). The lack of blue colouration in wild-type *Arabidopsis thaliana,* indicated absence of GUS activity (Fig. 5A). Increasing concentrations of ABA, PEG and NaCl were used to check the GUS activity in transgenic plants. Light colouration was observed in T_2_ plants harbouring the CaMV35S promoter construct, indicating low levels of GUS expression under ABA, PEG, and NaCl treatment (Fig. 5B). T_2_ plants with PS promoter showed a comparatively more intense blue coloration in roots and internodes, with high levels of GUS expression under NaCl (200 mM) treatment (Fig. 5C).

#### Relative *gus* expression analysis by qRT PCR

Transgenic T2 *Arabidopsis thaliana* plants treated with ABA (100 μM, 150 μM and 200 μM), NaCl (75 mM, 100 mM, 150 mM), or PEG (50 mM,100 mM and 200 mM) were harvested at 0, 12, 24, 48 h for relative *gus* expression analysis by qRT PCR. The experiment was performed using three biological triplicates of each treatment and time interval.

**Figure 6:**
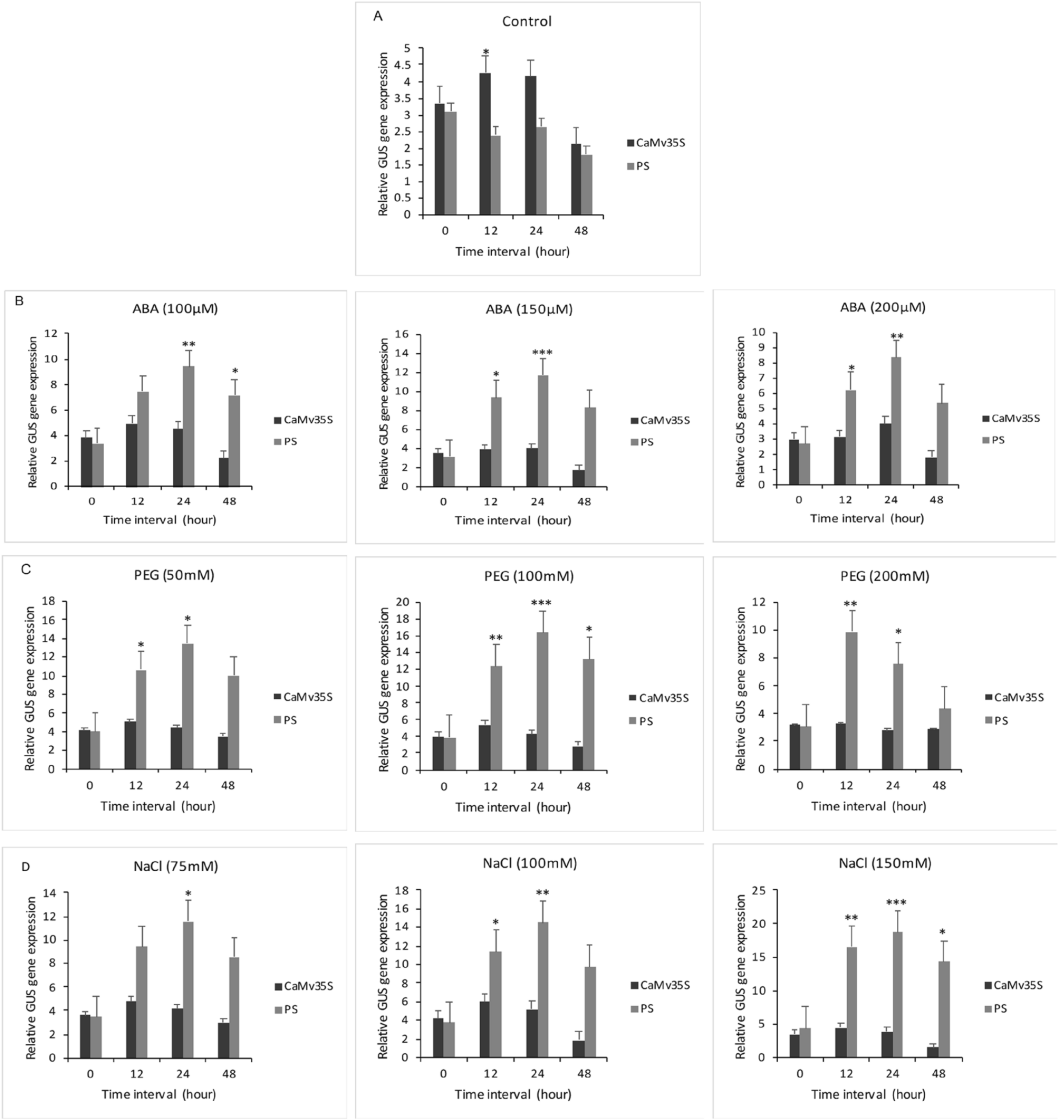
*gus* expression analysis of PS and CaMV 35S promoters under NaCl, PEG, and ABA stress. T2 transgenics were treated with different stress conditions and harvested at 0, 12, 24, and 48 h. (**A**) Control-*gus* expression levels of PS and CaMV35S transgenics in untreated conditions; (**B**) PS and CaMV35S transgenics treated with ABA (100 µM, 150 µM, 200 µM); (**C**) PS and CaMV35S transgenics treated with PEG (50 mM, 100 mM, 200 mM); (**D**) PS and CaMV35S transgenics treated with NaCl (75 mM, 100 mM, 150 mM). qRT-PCR data was normalized using the *ACT2* gene. A representative histogram with standard error from three replicates performed for each time point has been represented. The data represents the mean ± SD of three independent biological replicates; the asterisk (*P < 0.05; **P < 0.01; ***P < 0.001) indicates statistical significance calculated using the student’s *t*-test.

Control (no treatment) plants showed the results as shown (Fig. 6A).Under 150 μM ABA treatment, *gus* expression by PS was highest, more than two-fold compared to CaMV35S, at the 24-h interval (Fig. 6B). The expression levels decreased with higher concentration of ABA and longer time intervals (48 h). In the case of PEG, the highest activity of PS promoter (a 2.5-fold increase in *gus* induction compared to CaMV35S) was observed at a concentration of 100 mM at the 24-h interval. The *gus* induction levels by both constructs decreased at higher PEG concentrations and longer time intervals (Fig. 6C). *gus* was strongly induced by the PS promoter on treatment with different concentrations of NaCl (Fig. 6D). An approximately five-fold increase in *gus* expression was observed at the 24-h interval in comparison to CaMV35S, under 100 and 150 mM NaCl (Fig. 6D). The expression levels under all NaCl concentrations decreased at 48 h. We conclude that the designed PS promoter shows higher gene expression levels compared to the constitutive CaMV35S promoter, under all tested abiotic stress conditions.

## Discussion

The use of a constitutive promoter in expressing stress-responsive proteins is a metabolic burden for the transgenics and can potentially lead to unfavourable growth impact and decrease in productivity^40,41^. Studies have used subdomains of promoters such as the core sequence and their native surroundings to construct chimeric transcriptomic modules that occasionally resulted in higher activity than the parent promoter^42^. Constitutive gene expression does not depend on the stable presence of any particular transcription factor (TF), rather on the use of a broad variety of TFs. By incorporating an array of CREs from monocot and dicot species that function in salt-stress induction, we attempted to manually design a broad-range high activity salt-inducible promoter.

From a total of 2500 salinity-stress inducible genes, 2360 promoters were screened for high- occurrence CREs. A 454 bp module was designed based on the position, copy number, spacer distance, and frequency of CREs present in these promoters. Some of the incorporated CREs are also responsible for regulating stresses other than salinity stress (overlapping elements). For transcription initiation, elements like TATA and CAAT boxes were also introduced. Compared with the native promoter CaMV35S, the designed promoter PS has a higher number of CREs responsible for induction under salinity stress. Transient expression studies were conducted in *Nicotiana tabacum* leaves treated with different abiotic and hormonal stressors and harvested at 0, 12-, 24- and 48-h. In untreated conditions, the PS promoter showed similar levels of GUS activity to the CaMV35S promoter. The GUS activity increased by one-fold and 1.5-fold under jasmonic acid and salicylic acid in the case of PS promoter. The difference in GUS activity for PS promoter in comparison to CaMV35S under NaCl (seven-fold), ABA (four-fold), and PEG (five-fold) was significant at 24 h. The slight decrease in GUS activity at 48 h can be attributed to toxic accumulation or energy deprivation in the plant system. To validate the transient expression results, stable transgenic *Arabidopsis thaliana* plants were generated*.* GUS histochemical assay of transgenic T2 plants with PS promoter showed a deep blue colour when treated with 200 mM NaCl. The blue colour was qualitatively less intense in the case of T2 plants containing the CaMV35S promoter, indicating that the PS promoter induced GUS expression at higher levels under salinity stress. Real-time quantitative analysis revealed that the PS promoter induced *gus* gene expression by more than fivefold when treated with NaCl at the 24-h interval, compared to the CaMV35S promoter. A more than twofold increase in *gus* expression was observed under 150 μM ABA and 100 mM PEG treatment at the 24-h interval. The results show that the synthesized PS promoter induced gene expression at high levels under salt, drought, and ABA stress. However, a basal-level expression equivalent to CaMV35S promoter was also observed in untreated conditions. Deletion assays can help identify crucial elements responsible for the low-levels of induction in unstressed conditions.

To identify the essential processes or networks that underlie salinity tolerance, a comprehensive strategy is necessary. Despite substantial advances in our understanding of plant signalling pathways, there is still much to learn about sensors and receptors in signal transduction, transmembrane ion transport, metabolites in energy supply, and molecules in long-distance signalling. Future efforts should focus on understanding the molecular connections between and within cells contributing to the salt-stress response. Genetic engineering can create stress-tolerant plant varieties. This approach will be more effective when additional genes functioning in salt-tolerance are found and widely employed.

### Supplementary Information

Supplementary Table S1.

Supplementary Table S2.

Supplementary Legends.

## Data Availability

Data has been provided as a supplementary file; additional data will be provided on request to Prof. Rajesh Mehrotra. (rajeshm@goa.bits-pilani.ac.in).
