# Supplementary material for: Artificially designed synthetic promoter for a high level of salt induction using a cis-engineering approach
Source: Sci Rep. 2024 Jun 13;14:13657. doi: 10.1038/s41598-024-64537-z (PMC11176371; doi:10.1038/s41598-024-64537-z)
Supplement: Supplementary file 3 — Supplementary Legends. [file 41598_2024_64537_MOESM3_ESM.docx]

**Supplementary Files:**

1: List of genes up-regulated under salinity stress.

2: List of plants selected for synthesis of the designer promoter.
